# Exploring Genetic Information with Ease: The Linkout Plugin for JBrowse 2

**DOI:** 10.17912/micropub.biology.000906

**Published:** 2023-08-19

**Authors:** Chi-Hsien Chang, Szu-Ping Chen, Monica Poelchau, Christopher Childers

**Affiliations:** 1 Department of Electrical Engineering, National Taiwan University; 2 Institute of Agronomy, National Taiwan University; 3 USDA, Agricultural Research Service, National Agricultural Library

## Abstract

JBrowse 2 is a next-generation genome browser that can be run as a web or desktop application. We describe a new plugin, the Linkout Plugin, that enables users to link features to external databases based on their IDs and the remote URLs on JBrowse 2 desktop or web. As a result, genome analysis time and effort are reduced, enabling researchers to gain insights more quickly. The Linkout Plugin fills a common need scientists have: looking for more information on a gene. Overall, the Linkout Plugin is a valuable and practical addition to the JBrowse functionality.

**Figure 1. The Linkout Plugin for JBrowse 2:**
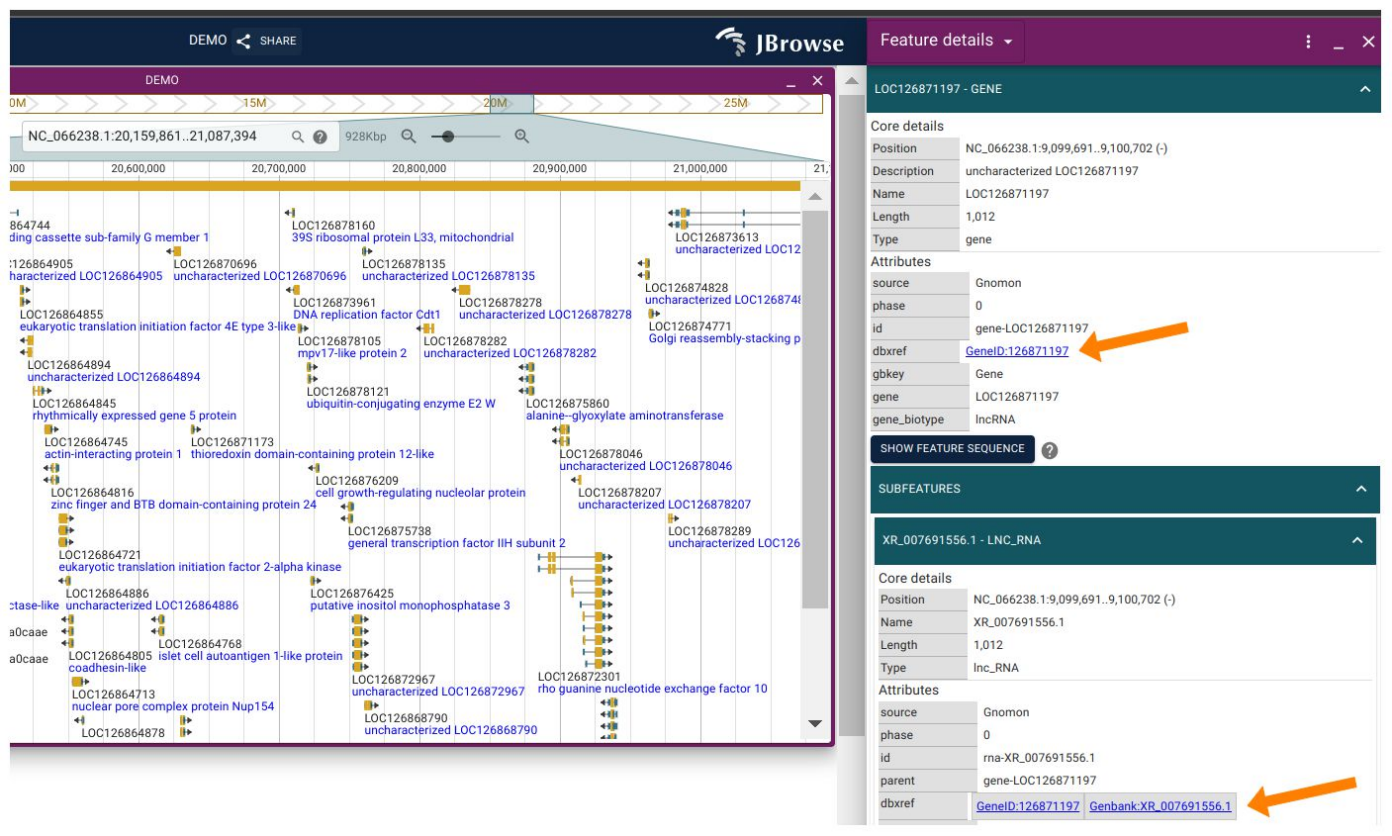
The Linkout Plugin is able to automatically add linkouts to the National Center for Biotechnology Information (NCBI) feature page if a feature has NCBI IDs on the JBrowse 2 desktop or web. Users can click the “GeneID:126871197” or “Genbank:XR_007691556.1” cross-references on the right-hand side of the dbxref section.

## Description

JBrowse 2 is a next-generation genome browser that can be run as a web or desktop application. Genome browsers serve as essential tools for efficient genome analysis. They enable researchers to visualize and explore genetic data while facilitating data retrieval and analysis. However, manual input of URLs to link out to related resources on feature pages can be time-consuming and inefficient. This led to the need for a tool that simplifies the process and improves user convenience.

We developed a plugin that allows users to easily configure hyperlinks to cross-references in the JBrowse data. Based on user-defined preferences, the Linkout Plugin automatically generates URLs in the JBrowse 2 feature details panel, which can point to external resources, such as National Center for Biotechnology Information (NCBI) or HUGO Gene Nomenclature Committee (HGNC) gene and nucleotide pages. Figure 1 shows adding link outs to the NCBI feature page if a feature has NCBI IDs on JBrowse 2 desktop or web. Once users click on "GeneID:126871197" or "Genbank:XR_007691556.1" in the dbxref on the right, the plugin will direct to the corresponding websites: https://www.ncbi.nlm.nih.gov/gene/126871197 or https://www.ncbi.nlm.nih.gov/nuccore/XR_007691556.1 . Users can easily configure hyperlinks to cross-references in the JBrowse data by simply installing the Linkout Plugin from the plugin store and inputting URL information in the Settings. A demo video is available for users to understand how to use the plugin: DEMO video .

With the Linkout Plugin, researchers can easily define where the generated URLs combined with the given gene IDs link to obtain genetic information. For example, researchers can define that linkouts to NCBI's feature page should be added automatically if a feature has NCBI or HGNC IDs within JBrowse 2. This plugin provides a direct connection to the external records, removing the need for manual searching and potential human error. This functionality reduces the time and effort involved in genome analysis, allowing researchers to focus on their analysis and gain insights more quickly. The Linkout Plugin fills a common need scientists have: looking for more information on a gene. Coupled with the simple configuration, this is a valuable new addition to JBrowse functionality.

The DOI of the Linkout Plugin is 10.5281/zenodo.8177361 . For user instructions, please refer to the Github tutorial at: https://github.com/NAL-i5K/jbrowse-plugin-linkout . Users can also obtain Linkout Plugin configuration in the Plugin Store at: https://jbrowse.org/jb2/plugin_store .

## Extended Data

Description: The demo vedio for the Linkout Plugin. Resource Type: Audiovisual. DOI: 10.22002/tg9a6-khw14

## References

[R1] Diesh Colin, Stevens Garrett J, Xie Peter, De Jesus Martinez Teresa, Hershberg Elliot A., Leung Angel, Guo Emma, Dider Shihab, Zhang Junjun, Bridge Caroline, Hogue Gregory, Duncan Andrew, Morgan Matthew, Flores Tia, Bimber Benjamin N., Haw Robin, Cain Scott, Buels Robert M., Stein Lincoln D., Holmes Ian H. (2023). JBrowse 2: a modular genome browser with views of synteny and structural variation. Genome Biology.

